# 3-Hy­droxy­methyl-1-(4-meth­oxy­phen­yl)imidazolidine-2,4-dione

**DOI:** 10.1107/S1600536810026838

**Published:** 2010-07-14

**Authors:** Xian-Chao Cheng, Jing-Jing Hou, Run-Ling Wang, Wei-Li Dong

**Affiliations:** a School of Pharmacy, Tianjin Medical University, Tianjin 300070, People’s Republic of China

## Abstract

In the title mol­ecule, C_11_H_12_N_2_O_4_, the dihedral angle between the benzene ring and imidazolidine ring is 7.1 (5)°. In the crystal structure, the hy­droxy groups are involved in the formation of inter­molecular O—H⋯O hydrogen bonds, which link the mol­ecules related by translation into *C*(2) chains along the *b* axis.

## Related literature

For related structures, see: Gerdil (1960 ▶); Sun *et al.* (2010 ▶). For details of the synthesis, see Niwata *et al.* (1997 ▶).
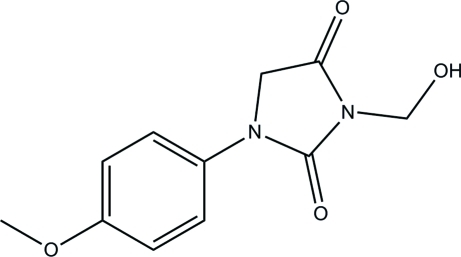


## Experimental

### 

#### Crystal data

C_11_H_12_N_2_O_4_
*M* *_r_* = 236.23
Monoclinic, 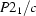

*a* = 21.280 (4) Å
*b* = 6.3309 (13) Å
*c* = 7.8813 (16) Å
β = 100.52 (3)°
*V* = 1043.9 (4) Å^3^
*Z* = 4
Mo *K*α radiation
μ = 0.12 mm^−1^
*T* = 113 K
0.20 × 0.18 × 0.12 mm

#### Data collection

Rigaku Saturn CCD area-detector diffractometer
Absorption correction: multi-scan (*CrystalClear*; Rigaku, 2005 ▶) *T*_min_ = 0.977, *T*_max_ = 0.986
7503 measured reflections
1841 independent reflections
1540 reflections with *I* > 2σ(*I*)
*R*_int_ = 0.042

#### Refinement

*R*[*F* ^2^ > 2σ(*F* ^2^)] = 0.039
*wR*(*F* ^2^) = 0.112
*S* = 1.09
1841 reflections
156 parameters
H-atom parameters constrained
Δρ_max_ = 0.18 e Å^−3^
Δρ_min_ = −0.23 e Å^−3^

### 

Data collection: *CrystalClear* (Rigaku, 2005 ▶); cell refinement: *CrystalClear*; data reduction: *CrystalClear*; program(s) used to solve structure: *SHELXTL* (Sheldrick, 2008 ▶); program(s) used to refine structure: *SHELXTL*; molecular graphics: *SHELXTL*; software used to prepare material for publication: *SHELXTL*.

## Supplementary Material

Crystal structure: contains datablocks I, global. DOI: 10.1107/S1600536810026838/cv2742sup1.cif

Structure factors: contains datablocks I. DOI: 10.1107/S1600536810026838/cv2742Isup2.hkl

Additional supplementary materials:  crystallographic information; 3D view; checkCIF report

## Figures and Tables

**Table 1:** Hydrogen-bond geometry (Å, °)

*D*—H⋯*A*	*D*—H	H⋯*A*	*D*⋯*A*	*D*—H⋯*A*
O3—H3⋯O4^i^	0.82	1.92	2.7346 (17)	174

Symmetry code: (i) 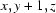.

**Fig. 1.**
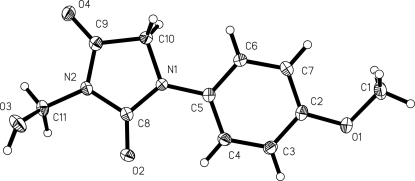
View of the title compound, with displacement ellipsoids drawn at the 40% probability level.

## supplementary crystallographic information

### Comment

During the research of novel antidiabetic agents, we found that imidazolidine-2,4-dione derivatives had potent antidiabetic activities. The crystal structure of the title compound was determined to investigate the relationship between structure and antidiabetic activity.

In the title compound, all bond lengths and angles are normal and in a good agreement with those reported previously (Gerdil, 1960; Sun *et al.*, 2010). The dihedral angle between the benzene ring (C2—C7) and imidazolidine ring (C9—C10/N1/N2) is 7.1 (5)°. In the crystal structure, the hydroxy groups are involved in formaton of intermolecular O—H···O hydrogen bonds (Table 1), which link the molecules related by translation along axis *b* into linear chains.

### Experimental

A mixture of 1-(4-methoxyphenyl)imidazolidine-2,4-dione (0.27 g, 1.32 mmol), 37% formaldehyde (2.1 ml, 27.9 mmol), and methanol (8 ml) was stirred at 70 ° C for 2 h. After the reaction, water (8 ml) was added and the precipitate was filtered and washed with water to give 3-(hydroxymethyl)-1-(4-methoxyphenyl)imidazolidine-2,4-dione (0.27 g, 90% yield) (Niwata *et al.*, 1997). Crystals suitable for X-ray diffraction were obtained through slow evaporation of a solution of the pure title compound in dichloromethane/methanol (1/1 by volume).

### Refinement

All H atoms were found on difference maps, with C—H = 0.95–0.99 Å and included in the final cycles of refinement using a riding model, with *U*_iso_(H) = 1.2*U*_eq_(C) for aryl and methylene H atoms and 1.5*U*_eq_(C,*O*) for the methyl and hydroxy H atoms.

### Crystal data

**Table:**

C_11_H_12_N_2_O_4_	*F*(000) = 496
*M**_r_* = 236.23	*D*_x_ = 1.503 Mg m^−^^3^
Monoclinic, *P*2_1_/*c*	Mo *K*α radiation, λ = 0.71073 Å
Hall symbol: -P 2ybc	Cell parameters from 2988 reflections
*a* = 21.280 (4) Å	θ = 2.0–27.9°
*b* = 6.3309 (13) Å	µ = 0.12 mm^−^^1^
*c* = 7.8813 (16) Å	*T* = 113 K
β = 100.52 (3)°	Platelet, colorless
*V* = 1043.9 (4) Å^3^	0.20 × 0.18 × 0.12 mm
*Z* = 4	

### Data collection

**Table:**

Rigaku Saturn CCD area-detector diffractometer	1841 independent reflections
Radiation source: rotating anode	1540 reflections with *I* > 2σ(*I*)
confocal	*R*_int_ = 0.042
Detector resolution: 7.31 pixels mm^-1^	θ_max_ = 25.0°, θ_min_ = 2.0°
ω and φ scans	*h* = −25→23
Absorption correction: multi-scan (*CrystalClear*; Rigaku, 2005)	*k* = −7→7
*T*_min_ = 0.977, *T*_max_ = 0.986	*l* = −7→9
7503 measured reflections	

### Refinement

**Table:**

Refinement on *F*^2^	Primary atom site location: structure-invariant direct methods
Least-squares matrix: full	Secondary atom site location: difference Fourier map
*R*[*F*^2^ > 2σ(*F*^2^)] = 0.039	Hydrogen site location: inferred from neighbouring sites
*wR*(*F*^2^) = 0.112	H-atom parameters constrained
*S* = 1.09	*w* = 1/[σ^2^(*F*_o_^2^) + (0.0696*P*)^2^ + 0.0281*P*] where *P* = (*F*_o_^2^ + 2*F*_c_^2^)/3
1841 reflections	(Δ/σ)_max_ < 0.001
156 parameters	Δρ_max_ = 0.18 e Å^−^^3^
0 restraints	Δρ_min_ = −0.23 e Å^−^^3^

### Special details

**Table:**

Geometry. All e.s.d.'s (except the e.s.d. in the dihedral angle between two l.s. planes) are estimated using the full covariance matrix. The cell e.s.d.'s are taken into account individually in the estimation of e.s.d.'s in distances, angles and torsion angles; correlations between e.s.d.'s in cell parameters are only used when they are defined by crystal symmetry. An approximate (isotropic) treatment of cell e.s.d.'s is used for estimating e.s.d.'s involving l.s. planes.
Refinement. Refinement of *F*^2^ against ALL reflections. The weighted *R*-factor *wR* and goodness of fit *S* are based on *F*^2^, conventional *R*-factors *R* are based on *F*, with *F* set to zero for negative *F*^2^. The threshold expression of *F*^2^ > σ(*F*^2^) is used only for calculating *R*-factors(gt) *etc*. and is not relevant to the choice of reflections for refinement. *R*-factors based on *F*^2^ are statistically about twice as large as those based on *F*, and *R*- factors based on ALL data will be even larger.

### Fractional atomic coordinates and isotropic or equivalent isotropic displacement parameters (Å^2^)

**Table:**

	*x*	*y*	*z*	*U*_iso_*/*U*_eq_	
O1	0.06206 (5)	0.43299 (18)	0.14946 (13)	0.0262 (3)	
O2	0.31479 (5)	0.92240 (17)	0.56402 (13)	0.0239 (3)	
O3	0.41891 (6)	0.98695 (18)	0.93286 (14)	0.0310 (3)	
H3	0.4247	1.1116	0.9119	0.046*	
O4	0.43355 (5)	0.41010 (18)	0.88227 (13)	0.0263 (3)	
N1	0.29378 (6)	0.5666 (2)	0.60155 (15)	0.0189 (3)	
N2	0.38408 (6)	0.7058 (2)	0.74760 (15)	0.0198 (3)	
C1	0.02888 (8)	0.2377 (3)	0.1525 (2)	0.0333 (4)	
H1A	0.0536	0.1259	0.1151	0.050*	
H1B	−0.0118	0.2463	0.0767	0.050*	
H1C	0.0225	0.2095	0.2679	0.050*	
C2	0.11895 (7)	0.4547 (3)	0.26381 (19)	0.0207 (4)	
C3	0.14827 (7)	0.6511 (3)	0.26585 (19)	0.0229 (4)	
H3A	0.1292	0.7568	0.1921	0.027*	
C4	0.20547 (7)	0.6918 (3)	0.37598 (19)	0.0216 (4)	
H4	0.2243	0.8244	0.3768	0.026*	
C5	0.23490 (7)	0.5332 (3)	0.48603 (18)	0.0188 (4)	
C6	0.20577 (7)	0.3369 (3)	0.48223 (18)	0.0208 (4)	
H6	0.2252	0.2302	0.5544	0.025*	
C7	0.14802 (7)	0.2966 (3)	0.3726 (2)	0.0234 (4)	
H7	0.1290	0.1644	0.3722	0.028*	
C8	0.32780 (7)	0.7496 (2)	0.62781 (19)	0.0188 (4)	
C9	0.38857 (7)	0.4969 (3)	0.78904 (18)	0.0208 (4)	
C10	0.32821 (7)	0.3927 (3)	0.69862 (18)	0.0208 (4)	
H10A	0.3374	0.2813	0.6222	0.025*	
H10B	0.3041	0.3342	0.7807	0.025*	
C11	0.43450 (7)	0.8604 (3)	0.79990 (19)	0.0237 (4)	
H11A	0.4748	0.7887	0.8400	0.028*	
H11B	0.4392	0.9482	0.7021	0.028*	

### Atomic displacement parameters (Å^2^)

**Table:**

	*U*^11^	*U*^22^	*U*^33^	*U*^12^	*U*^13^	*U*^23^
O1	0.0191 (6)	0.0278 (7)	0.0292 (6)	−0.0026 (5)	−0.0022 (5)	−0.0004 (5)
O2	0.0255 (6)	0.0176 (6)	0.0282 (6)	−0.0021 (5)	0.0038 (5)	0.0026 (5)
O3	0.0468 (8)	0.0223 (7)	0.0258 (6)	−0.0109 (6)	0.0117 (5)	−0.0066 (5)
O4	0.0242 (6)	0.0259 (7)	0.0264 (6)	0.0028 (5)	−0.0021 (5)	−0.0016 (5)
N1	0.0182 (7)	0.0165 (7)	0.0207 (7)	−0.0004 (5)	0.0000 (5)	0.0008 (5)
N2	0.0198 (7)	0.0196 (7)	0.0196 (7)	−0.0036 (5)	0.0026 (5)	−0.0025 (5)
C1	0.0251 (9)	0.0371 (11)	0.0347 (9)	−0.0111 (8)	−0.0024 (7)	0.0010 (8)
C2	0.0170 (8)	0.0262 (9)	0.0189 (8)	0.0003 (6)	0.0029 (6)	−0.0035 (7)
C3	0.0215 (8)	0.0233 (9)	0.0234 (8)	0.0019 (7)	0.0028 (6)	0.0033 (7)
C4	0.0213 (8)	0.0189 (8)	0.0247 (8)	−0.0008 (6)	0.0046 (6)	0.0014 (7)
C5	0.0180 (8)	0.0214 (8)	0.0175 (8)	−0.0004 (6)	0.0048 (6)	−0.0021 (6)
C6	0.0207 (8)	0.0195 (9)	0.0216 (8)	0.0005 (6)	0.0024 (6)	0.0019 (6)
C7	0.0231 (8)	0.0208 (9)	0.0262 (8)	−0.0049 (7)	0.0040 (6)	−0.0017 (7)
C8	0.0188 (8)	0.0205 (8)	0.0182 (8)	−0.0021 (6)	0.0063 (6)	−0.0023 (6)
C9	0.0224 (8)	0.0222 (9)	0.0182 (8)	0.0012 (7)	0.0049 (6)	−0.0027 (6)
C10	0.0222 (8)	0.0181 (8)	0.0211 (8)	0.0004 (6)	0.0017 (6)	−0.0001 (6)
C11	0.0206 (8)	0.0267 (9)	0.0235 (8)	−0.0069 (7)	0.0032 (6)	−0.0034 (7)

### Geometric parameters (Å, °)

**Table:**

O1—C2	1.3778 (18)	C2—C7	1.388 (2)
O1—C1	1.426 (2)	C2—C3	1.390 (2)
O2—C8	1.2147 (19)	C3—C4	1.384 (2)
O3—C11	1.406 (2)	C3—H3A	0.9300
O3—H3	0.8200	C4—C5	1.398 (2)
O4—C9	1.2252 (19)	C4—H4	0.9300
N1—C8	1.3614 (19)	C5—C6	1.387 (2)
N1—C5	1.4238 (19)	C6—C7	1.391 (2)
N1—C10	1.460 (2)	C6—H6	0.9300
N2—C9	1.361 (2)	C7—H7	0.9300
N2—C8	1.411 (2)	C9—C10	1.503 (2)
N2—C11	1.4556 (19)	C10—H10A	0.9700
C1—H1A	0.9600	C10—H10B	0.9700
C1—H1B	0.9600	C11—H11A	0.9700
C1—H1C	0.9600	C11—H11B	0.9700
			
C2—O1—C1	117.01 (13)	C4—C5—N1	122.16 (14)
C11—O3—H3	109.5	C5—C6—C7	121.23 (15)
C8—N1—C5	127.24 (13)	C5—C6—H6	119.4
C8—N1—C10	111.11 (12)	C7—C6—H6	119.4
C5—N1—C10	121.46 (13)	C2—C7—C6	119.68 (15)
C9—N2—C8	111.44 (12)	C2—C7—H7	120.2
C9—N2—C11	124.73 (13)	C6—C7—H7	120.2
C8—N2—C11	123.31 (13)	O2—C8—N1	128.95 (14)
O1—C1—H1A	109.5	O2—C8—N2	123.76 (14)
O1—C1—H1B	109.5	N1—C8—N2	107.30 (13)
H1A—C1—H1B	109.5	O4—C9—N2	126.35 (15)
O1—C1—H1C	109.5	O4—C9—C10	126.42 (16)
H1A—C1—H1C	109.5	N2—C9—C10	107.23 (12)
H1B—C1—H1C	109.5	N1—C10—C9	102.77 (13)
O1—C2—C7	124.84 (15)	N1—C10—H10A	111.2
O1—C2—C3	115.85 (14)	C9—C10—H10A	111.2
C7—C2—C3	119.31 (15)	N1—C10—H10B	111.2
C4—C3—C2	121.01 (15)	C9—C10—H10B	111.2
C4—C3—H3A	119.5	H10A—C10—H10B	109.1
C2—C3—H3A	119.5	O3—C11—N2	109.40 (12)
C3—C4—C5	119.91 (15)	O3—C11—H11A	109.8
C3—C4—H4	120.0	N2—C11—H11A	109.8
C5—C4—H4	120.0	O3—C11—H11B	109.8
C6—C5—C4	118.84 (14)	N2—C11—H11B	109.8
C6—C5—N1	119.00 (14)	H11A—C11—H11B	108.2
			
C1—O1—C2—C7	4.5 (2)	C10—N1—C8—O2	177.46 (15)
C1—O1—C2—C3	−176.06 (14)	C5—N1—C8—N2	−177.40 (13)
O1—C2—C3—C4	179.71 (13)	C10—N1—C8—N2	−2.44 (17)
C7—C2—C3—C4	−0.8 (2)	C9—N2—C8—O2	−175.85 (14)
C2—C3—C4—C5	0.7 (2)	C11—N2—C8—O2	−3.8 (2)
C3—C4—C5—C6	0.0 (2)	C9—N2—C8—N1	4.06 (17)
C3—C4—C5—N1	179.89 (14)	C11—N2—C8—N1	176.11 (12)
C8—N1—C5—C6	−177.26 (14)	C8—N2—C9—O4	175.87 (14)
C10—N1—C5—C6	8.2 (2)	C11—N2—C9—O4	4.0 (2)
C8—N1—C5—C4	2.8 (2)	C8—N2—C9—C10	−3.93 (17)
C10—N1—C5—C4	−171.66 (14)	C11—N2—C9—C10	−175.84 (12)
C4—C5—C6—C7	−0.5 (2)	C8—N1—C10—C9	0.15 (15)
N1—C5—C6—C7	179.56 (14)	C5—N1—C10—C9	175.45 (13)
O1—C2—C7—C6	179.69 (13)	O4—C9—C10—N1	−177.52 (15)
C3—C2—C7—C6	0.2 (2)	N2—C9—C10—N1	2.27 (15)
C5—C6—C7—C2	0.4 (2)	C9—N2—C11—O3	−105.04 (16)
C5—N1—C8—O2	2.5 (3)	C8—N2—C11—O3	83.98 (17)

### Hydrogen-bond geometry (Å, °)

**Table:**

*D*—H···*A*	*D*—H	H···*A*	*D*···*A*	*D*—H···*A*
O3—H3···O4^i^	0.82	1.92	2.7346 (17)	174

Symmetry codes: (i) *x*, *y*+1, *z*.
